# A Real-World Experience of Hyperkalemia Management Using Sodium Zirconium Cyclosilicate in Chronic Hemodialysis: A Multicenter Clinical Audit

**DOI:** 10.7759/cureus.45058

**Published:** 2023-09-11

**Authors:** XiaoJie Qu, Yan Hua, Behram A Khan

**Affiliations:** 1 Medical Affairs, The National Kidney Foundation Singapore, Singapore, SGP; 2 Nephrology, National University of Singapore, Singapore, SGP

**Keywords:** sodium zirconium cyclosilicate, asian patients, intradialytic, potassium binders, normokalemia, hemodialysis complication, international and travel medicine, dialysis center, chronic hemodialysis, severe hyperkalemia

## Abstract

Introduction: Hyperkalemia, a common condition among hemodialysis (HD) patients, is associated with adverse health outcomes. Evidence of the safety and efficacy of a potassium-binder, sodium zirconium cyclosilicate (SZC), has been limited among Asian (HD) patients beyond phase 3 trials. This article demonstrates real-world evidence of SZC usage in an Asian cohort of HD patients.

Methods: A retrospective clinical audit was conducted among 293 patients who received maintenance HD at community-based dialysis centers in Singapore. Patients received SZC for either management of hyperkalemia or hyperkalemia prevention during anticipated disruption to dialysis, such as during traveling. Among patients treated for hyperkalemia (N = 147), serum potassium (K+) prior to SZC initiation and at the endpoint was compared using a paired Student’s t-test. Changes in K+ from baseline to endpoint were compared across various categories within each demographic and health-related variables using either Student’s t-test or one-way ANOVA. Patients who experienced adverse events after SZC initiation or were deceased during the audit were reviewed to provide a descriptive account.

Results: Among patients who received SZC for hyperkalemia treatment, SZC use was associated with a significant reduction of 0.812 mmol/L in serum potassium. Patients with ethnicities other than Chinese, Malay, or Indian had a nominal reduction in K+ of 0.7 mmol/L and this can be accounted for the small sample size of this sub-group. The three main ethnicities which represented more than 95% of the sample showed a significant reduction in K+ levels (all three p<0.001). This is consistent with other studies with SZC which showed efficacy across various ethnicities. Patients who received SZC for hyperkalemia treatment or prevention had a significant lowering of mortality rate. This mortality reduction may have inherent biases and confounders, due to the retrospective clinical audit study design.

Conclusions: Overall, SZC was safe and effective among the audited patients. The efficacy in the real-world setting was similar to previous trials. The novel use of SZC to manage serum potassium when HD sessions are missed, such as during traveling, warrants further investigation due to potentially significant life-saving implications.

## Introduction

With the increased incidence of end-stage renal diseases (ESRD), more patients are treated with hemodialysis (HD) in Singapore [1]. From year 2011 to 2021, the annual incidence number of patients placed on HD has increased from 965 to 1297 [1]. 

Hyperkalemia, defined as a serum potassium level higher than 5 mmol/L, is a common condition among HD patients [2,3]. It is associated with an increased risk of all-cause mortality and hospitalization [4,5]. This has been observed in HD patients in Singapore in recent years, even during the COVID-19 pandemic, with its associated challenges in clinical management [6,7]. Furthermore, HD being a costly treatment modality for ESRD, requires measures to reduce complications associated with it, to reduce the financial burden on the patient and the healthcare system [8]. 

Dietary restriction, use of low-potassium dialysate, discontinuation of certain medications, and treatment with diuretics or potassium binders are some of the viable options for the management of hyperkalemia [9,10]. Sodium polystyrene sulfonate (SPS) and sodium zirconium cyclosilicate (SZC) are the two potassium binders available in Singapore. The efficacy of these potassium binders has been evaluated in clinical trials [11-14]. However, there have been concerns regarding the safety of SPS. The US Food and Drug Administration has issued a warning for colonic necrosis and other serious gastrointestinal (GI) adverse events associated with SPS use [15]. SZC, a newer and novel potassium binder, has been shown to be effective and well-tolerated for the treatment of hyperkalemia in patients with ESRD receiving adequate HD [16].

Evidence of the safety and efficacy of a potassium binder, SZC, has been limited among Asian (HD) patients beyond phase 3 trials and initial studies [11-14]. This article demonstrates real-world evidence of SZC usage in an Asian cohort of HD patients in Singapore.

## Materials and methods

Study design and subjects

The study design is a retrospective clinical audit, derived from routine clinical treatments provided to patients. Such a study design is exempt from Institutional Review Board approvals. The study team used aggregated data sets, with de-identified patient data. 

The patients received their chronic HD treatments at 42 community-based HD centers located island-wide in Singapore, with their acute HD treatments (if hospitalized) at various public hospitals. SZC was introduced to the list of permitted medications in the chronic HD centers in May 2021. A retrospective clinical audit was conducted among these patients who received SZC between May 2021 and January 2023 as part of their chronic HD program management to evaluate the effectiveness of SZC in hyperkalemia management and prevention among an Asian cohort.

Data collection

The retrospective clinical audit was done as a one-time exercise, with the refinement of audit parameters based on the study team's requirements. These refinements were done by the medical affairs team of the dialysis provider, as part of clinical care quality review, with any additional queries from the study team. The audited patients were separated into two groups based on the indication for SZC use. Patients in Group 1 received SZC for management of hyperkalemia and those in Group 2 received SZC for hyperkalemia prevention during traveling. Both groups received routine and identical standards of care, including general measures for hyperkalemia control prior to SZC usage, such as dietary counseling and management for low potassium dialysate levels of 2 mEq/L. As this was not a prospective trial, such measures could not be additionally controlled for in this retrospective clinical audit. The Group 2 patients were exposed to increased inter-dialytic times due to missed or delayed dialysis, due to their travel plans. Appropriate counseling regarding increased risk of delayed or missed dialysis was done for Group 2 patients, as part of their routine clinical care, with "Against Ordered Recommendation" forms signed to confirm their understanding. The dose and duration of SZC prescribed were based on the attending nephrologist's discretion.

Information on patients’ demographic and health-related variables was obtained from a clinical dashboard, designed to extract aggregated and de-identified data from the electronic medical records. These variables were analyzed as categorical variables according to the categories listed in Table 1 in the results section.

Statistical analyses

For patients in Group 1, potassium level prior to SZC initiation (i.e., baseline) and at the endpoint was summarized and compared using paired Student’s t-test. The endpoint is defined as 31st January 2023 or patients’ date of death, whichever came earlier. Group 1 patients who did not have a serum potassium measurement after SZC initiation were excluded from the analysis. Changes in serum potassium from baseline to endpoint were compared across various categories within each demographic and health-related variables. For binary variables, a comparison was made using an independent Student’s t-test. For variables with multiple categories, a one-way ANOVA test was conducted. The percentage of patients achieving normal serum potassium levels (defined as 3.5 mmol/L to 5 mmol/L) at the endpoint was calculated. 

All patients (in both Groups 1 and 2) who experienced any adverse events after SZC initiation or were deceased during the audit period were reviewed to provide a descriptive account of the findings. Statistical significance was defined as p-value < 0.05. All statistical analysis was performed using IBM SPSS statistics for Windows version 26.0 software (IBM Corp., Armonk, NY).

## Results

Characteristics of participants

Over the audit period of 19 months, 293 patients were prescribed SZC. Among these patients, 147 received SZC for the treatment of hyperkalemia (Group 1) and 146 received SZC for hyperkalemia prevention during traveling (Group 2). Details of patients’ demographic and health-related variables are presented in Table 1. 

**Table 1:** Characteristics of patients who were treated with sodium zirconium cyclosilicate Number of patients were summarized as count (percentage). IQR: interquartile range. SD: standard deviation. GN: glomerulonephritis. N: count. SZC: sodium zirconium cyclosilicate. ESRD: end-stage renal disease. HD: hemodialysis.

Characteristics	Total (N=293)	Group 1 (N=147)	Group 2 (N=146)
Age (Years)			
Median (IQR)	63.0 (56.0, 70.0)	66.0 (58.0, 73.0)	60.0 (54.0, 68.0)
<=50	39 (13.3%)	10 (6.8%)	29 (19.9%)
51-60	79 (27.0%)	34 (23.1%)	45 (30.8%)
61-70	104 (35.5%)	53 (36.1%)	51 (34.9%)
71-80	58 (19.8%)	42 (28.6%)	16 (11.0%)
>80	13 (4.4%)	8 (5.4%)	5 (3.4%)
Gender			
Female	133 (45.4%)	75 (51.0%)	58 (39.7%)
Male	160 (54.6%)	72 (49.0%)	88 (60.3%)
Ethnicity			
Chinese	151 (51.5%)	75 (51.0%)	76 (52.1%)
Indian	31 (10.6%)	18 (12.2%)	13 (8.9%)
Malay	97 (33.1%)	44 (29.9%)	53 (36.3%)
Others	14 (4.8%)	10 (6.8%)	4 (2.7%)
Dialysis vintage (duration since HD initiation)			
Median (IQR)	5.83 (3.36, 9.56)	6.43 (4.15, 9.92)	4.98 (2.69, 8.84)
<= 1 year	11 (3.8%)	2 (1.4%)	9 (6.2%)
1-3 years	56 (19.1%)	24 (16.3%)	32 (21.9%)
>3 years	226 (77.1%)	121 (82.3%)	105 (71.9%)
Primary etiology of ESRD			
Diabetes mellitus	149 (50.9%)	79 (53.7%)	70 (47.9%)
GN/presumed GN	70 (23.9%)	33 (22.4%)	37 (25.3%)
Hypertension	39 (13.3%)	16 (10.9%)	23 (15.8%)
Others	35 (11.9%)	19 (12.9%)	16 (11.0%)
Duration on SZC (months)			
Mean ± SD	2.13 ± 3.31	4.11 ± 3.74	0.131 ± 0.182
Baseline potassium (mmol/L)			
Mean ± SD	5.46 ± 0.903	6.14 ± 0.490	4.77 ± 0.666

The majority of the patients prescribed with SZC were males (N = 160, 54.6%) and of Chinese ethnicity (N = 151, 51.5%). The median age of the patients was 63 (interquartile range (IQR) 56.0-70.0 years). In total, 11 patients (3.8%) had dialysis vintage of one year or less. More than half (N = 149, 50.9%) of the patients had diabetes mellitus (DM) as their primary diagnosis. The mean SZC treatment duration was 2.13 ± 3.31 months. The mean baseline K+ for patients treated for hyperkalemia (Group 1) was 6.14 ± 0.490 mmol/L. All patients with hyperkalemia received their HD with a 2 mEq/L potassium dialysate. It was also seen that there were no additional measures taken to change the dialysate prescription or to alter the Kt/V as measures to control hyperkalemia.

Outcomes

After excluding 19 patients who did not have K+ measurement after SZC initiation, a total of 128 patients from Group 1 were included in the analysis of K+ levels. Overall, patients’ mean K+ was reduced by 0.812 mmol/L (95% confidence interval (CI) 0.691 mmol/L, 0.932 mmol/L, p<0.001) from baseline to endpoint. In total, 44 patients (34.4%) achieved normal K+ levels at the endpoint. Changes in K+ levels across various categories of demographic and health-related variables are summarized in Table 2. 

**Table 2:** Change in serum potassium levels from baseline to endpoint among patients with hyperkalemia Significant p-value denoted by asterisk(s) in the legend. *p-value <0.05 (obtained from paired Student’s t-test when patients’ endpoint and baseline potassium were compared). **p-value <0.001 (obtained from paired Student’s t-test when patients’ endpoint and baseline potassium were compared). ^1^ P-value for comparison refers to p-values obtained when a change in serum potassium from baseline to endpoint was compared across the various categories of patients’ demographic or health-related variables CI: confidence interval; GN: glomerulonephritis; N: count; SD: standard deviation; K+: serum potassium; ESRD: end-stage renal disease

Characteristic	N	Baseline K+ level (mean ± SD)	Endpoint K+ level (mean ± SD)	Differences in K+ level. Estimate (95%CI).	P-value for comparison^1 ^
Overall	128 (100%)	6.14 ± 0.488	5.32 ± 0.658	0.812 (0.691, 0.932)**	
Age (Years)					0.432
<=50	10 (7.8%)	6.23 ± 0.589	5.46 ± 0.55	0.770 (0.188, 1.35)*	
51-60	30 (23.4%)	6.23 ± 0.529	5.34 ± 0.762	0.893 (0.635, 1.15)**	
61-70	41 (32.0%)	6.08 ± 0.519	5.37 ± 0.56	0.717 (0.500, 0.934)**	
71-80	39 (30.5%)	6.14 ± 0.43	5.36 ± 0.699	0.779 (0.552, 1.01)**	
>80	8 (6.3%)	5.92 ± 0.231	4.72 ± 0.406	1.20 (0.857, 1.54)**	
Gender					0.776
Female	68 (53.1%)	6.21 ± 0.478	5.39 ± 0.693	0.828 (0.649, 1.01)**	
Male	60 (46.9%)	6.05 ± 0.488	5.26 ± 0.615	0.793 (0.629, 0.958)**	
Ethnicity					0.253
Chinese	66 (51.6%)	6.08 ± 0.561	5.23 ± 0.671	0.855 (0.662, 1.05)**	
Indian	15 (11.7%)	6.17 ± 0.366	5.15 ± 0.491	1.01 (0.745, 1.28)**	
Malay	38 (29.7%)	6.25 ± 0.41	5.52 ± 0.633	0.737 (0.555, 0.918)**	
Others	9 (7.0%)	6 ± 0.312	5.52 ± 0.769	0.478 (-0.0749, 1.03)	
Dialysis vintage	A meaningful comparison cannot be made as there was only one patient in the category of one year or less.				
Primary etiology of ESRD					0.705
Diabetes Mellitus	68 (53.1%)	6.12 ± 0.521	5.26 ± 0.642	0.857 (0.702, 1.01)**	
GN/Presumed GN	30 (23.4%)	6.06 ± 0.443	5.33 ± 0.617	0.730 (0.460, 1.00)**	
Hypertension	15 (11.7%)	6.25 ± 0.429	5.36 ± 0.773	0.893 (0.520, 1.27)**	
Others	15 (11.7%)	6.26 ± 0.473	5.57 ± 0.695	0.687 (0.207, 1.17)*	

It was found that patients’ age, gender, ethnicity, and primary etiology of ESRD were not associated with significant differences in K+ reduction. Three adverse events (AEs) associated with SZC administration were identified. All the AEs affected the GI tract; one patient experienced diarrhea (0.3%), one patient had abdominal pain (0.3%), and another experienced constipation (0.3%). None of the AEs necessitated immediate treatment or hospitalization. The AEs were therefore considered mild. Furthermore, none of the traveling patients reported AEs associated with SZC use.

In total, 10 patients (3.4%) died during the audit period of one year and nine months (May 2021 to Jan 2023) in both groups included in the study. Six of the deceased patients were from Group 1 and four were from Group 2 in the study. None of the deaths were reported due to hyperkalemia, cardiac arrhythmias, or cardiac arrest. Among the traveling patients included in the audit (Group 2), the crude mortality rate was 2.7% (N=4) and none of the traveling patients' cause of death was reported due to hyperkalemia, cardiac arrhythmias, or cardiac arrest. In comparison, the total number of patients receiving HD treatments in the same dialysis centers as the study group averaged around 4500 during the same audit period. In this comparative group, the overall crude mortality for these dialysis centers was recorded at around 14% for the same time frame. The study group therefore showed a reduction in mortality rate from 14% to 3.4%, with the addition of SZC usage. This is a reduction of 75.71% (p-value <0.01).

## Discussion

This real-world evidence from a retrospective clinical audit has demonstrated that SZC is effective in reducing serum potassium levels for the management of hyperkalemia among chronic HD patients undergoing dialysis in community-based dialysis centers. A mean reduction of 0.812 mmol/L in K+ was observed after treatment. This was consistent with clinical trials which showed that SZC resulted in a 0.46-1.28 mmol/L reduction in K+ levels within 48 hours after administration [11-14]. The proportion of patients who achieved normal K+ levels (33.4%) at the clinical audit endpoint was lower than that reported by other trials which reported that normal K+ levels were achieved in 41.2-99% of the patients after treatment with SZC [11-14]. This difference could potentially be explained by the longer audit duration of 19 months and the differences in patient population, as patients receiving dialysis were excluded from these comparative trials. Furthermore, some of our study patients did not receive SZC continuously due to either physicians’ clinical judgment or patients’ non-compliance. Thus, the benefits of SZC observed during our clinical audit may not be fully realized. Considering these findings, it would be reasonable to advocate that the treatment with a potassium binder alone may not be sufficient for optimal management of hyperkalemia in a real-world setting among chronic HD patients and other treatment options such as dietary potassium restriction should still be considered. 

Our clinical audit showed that SZC is effective in reducing K+ levels regardless of patients’ demographic background and primary etiology of ESRD. This observation was consistent with findings from clinical trials which demonstrated that SZC’s efficacy was not affected by age, gender, ethnicity, or comorbidity [12,16]. One exception in our study was patients with ethnicities other than Chinese, Malay, or Indian who experienced a nominal reduction of K+ levels. This could potentially be explained by the small sample size of this group of patients.

In addition to hyperkalemia management, SZC was also prescribed to patients traveling overseas for the prevention of hyperkalemia (Group 2 of our study). With missed or delayed dialysis sessions and increased risk of dietary non-compliance during traveling, is reasonable to assume that traveling patients may be at higher risk of hyperkalemia-related morbidity and mortality. Among the traveling patients included in our study, the crude mortality rate was 2.7% (N=4) and none of them had hyperkalemia, cardiac arrhythmias, or cardiac arrest as the cause of their death. Furthermore, none of the traveling patients reported AEs associated with SZC use, which is crucial to compliance especially when on holiday or during travel. This may suggest that a short course of SZC is safe for chronic HD patients who plan to travel. Conversely, it can be argued that patients who can travel may be healthier than the overall chronic HD patient cohort, thus contributing to the observed low mortality rate. The effect of SZC on reducing hyperkalemia-related mortality among traveling HD patients may be a novel use of potassium binders and warrants further study.

The proportion of audited patients with GI-adverse events associated with SZC (abdominal pain 0.3%, diarrhea 0.3%, and constipation 0.3%) was comparable to that reported in the literature (abdominal pain or distention 0.5%, diarrhea 0.9%, and constipation 2.9%) [14]. However, our clinical audit did not reveal other commonly reported AEs related to SZC use such as hypokalemia and edema [14]. These AEs may be more pertinent in patients who are not on chronic HD.

The significant reduction in mortality rate in the SZC study groups compared to the overall HD population in the same dialysis centers and during the same study period was an unexpected finding. As this was not a randomized clinical trial or a prospective study, this finding may have a number of inherent biases and confounders. Additionally, the general HD population may be demographically and clinically different from the study groups.

Strengths and limitations

This study’s main strength is the real-world evidence and reporting of using SZC in chronic HD patients, especially in an Asian cohort setting. Such literature can provide valuable practical usage and implementation insights to nephrologists practicing in busy outpatient chronic HD centers. The study also provided a novel usage of SZC in traveling patients, which may be useful in countering the effects of hyperkalemia in chronic HD patients in a setting that may delay dialysis or cause missed dialysis sessions for various reasons. The audit also addressed the constant potassium content in the dialysate and any measures to alter clearance (Kt/V), prior to initiation of SZC. The main limitation is the use of a control group of the general HD population in the same dialysis center, which may not be matched demographically or clinically to the study group. The study design is also not prospective and is not randomized, leading to the introduction of biases and confounders to interpret such comparative results. Lastly, due to the retrospective audit of patient records, some data points may be missing, which we are unable to remedy. 

## Conclusions

This real-world evidence from our clinical audit has demonstrated that the novel potassium binder, sodium zirconium cyclosilicate, was safe and effective in chronic hemodialysis patients. The adverse events reported were consistent with previous trials and were noted to be mild. It was effective in reducing hyperkalemia as a treatment in chronic HD setting. The findings were derived from studying an Asian cohort of patients being treated at a community-based hemodialysis centers in Singapore, and were consistent with previous literature supporting its usage across various ethnicities. The study revealed novel usage of SZC in patients at increased risk of hyperkalemia due to delayed or missed HD sessions, such as those traveling. Although our data showed a significant reduction in overall mortality by using SZC in this retrospective clinical audit, this finding needs to be further investigated by conducting prospective randomized controlled trials in chronic HD patients.
